# Effects of Dienogest Therapy on Endometriosis-Related Dysmenorrhea, Dyspareunia, and Endometrioma Size

**DOI:** 10.7759/cureus.34162

**Published:** 2023-01-24

**Authors:** Bilgehan Saglik Gokmen, Nura F Topbas Selcuki, Alev Aydın, Pinar Yalcin Bahat, Aysu Akça

**Affiliations:** 1 Obstetrics and Gynecology, Elazig Kovancilar State Hospital, Istanbul, TUR; 2 Obstetrics and Gynecology, University of Health Sciences Turkey, Istanbul Sisli Hamidiye Etfal Training and Research Hospital, Istanbul, TUR; 3 Obstetrics and Gynecology, University of Health Sciences Turkey, Istanbul Kanuni Sultan Suleyman Training and Research Hospital, Istanbul, TUR; 4 Infertility, Memorial Regional Hospital, Istanbul, TUR

**Keywords:** dyspareunia, dysmenorrhea, dienogest, endometriosis, endometrioma

## Abstract

Introduction: Endometriosis is an estrogen-dependent chronic inflammatory disease that is defined by the presence of endometrial-like tissue outside of the uterus. The most common localization is the ovaries, and endometriosis in this location is then called an endometrioma. According to the European Society of Human Reproduction and Embryology (ESHRE) (2022) guidelines, the most commonly prescribed treatments for endometriosis include drugs that alter the hormonal milieu. Dienogest is a new generation of progestin used in the treatment of endometriosis. The aim of this study was to assess the effect of Dienogest treatment on endometrioma size and endometriosis-related pain symptoms over a six-month follow-up period.

Methods: This prospective observational study was conducted at a tertiary clinic in Turkey between March 2020 and March 2021. Here, 64 patients aged 17-49 years with unilateral or bilateral endometriomas without any hormone-dependent cancers and any medical conditions contraindicating the onset of hormonal treatment, such as active venous thromboembolism, previous or current cardiovascular disease, diabetes with cardiovascular complications, current severe liver disease, and not being pregnant, were included. Endometrioma sizes were determined by transvaginal ultrasonography (TVUS). Dysmenorrhea and dyspareunia symptoms were evaluated using the visual analogue scale (VAS). Patients received Dienogest 2 mg/day continuously for six months. At the three- and six-month follow-ups, the patients were re-evaluated.

Results: The mean endometrioma size decreased significantly from an initial measurement of 44.0 ± 13 mm to 39.5 ± 15 mm at three months and to 34.4 ± 18 mm at the six-month follow-up. The mean dysmenorrhea VAS scores before treatment, at the three-month follow-up, and at the six-month follow-up were 6.9 ± 2.6, 4.3 ± 2.8, and 3.8 ± 2.7, respectively. Dysmenorrhea VAS scores decreased significantly over the first three months (p<0.01). Similarly, the mean VAS score for dyspareunia decreased at three and six months compared with the pretreatment value (p<0.01).

Conclusion: This study shows that dienogest treatment reduced the symptoms of dysmenorrhea and dyspareunia and the size of endometriomas. However, the main significant decrease in dysmenorrhea and dyspareunia symptoms was noted in the first three months, making it a good treatment option, especially in young patients with a fertility wish.

## Introduction

Endometriosis is an estrogen-dependent chronic inflammatory disease that is defined by the presence of endometrial-like tissue outside of the uterus [1]. The most common localization is the ovaries, which are then called endometriomas. It affects almost 10% of women who are in their reproductive years [2]. Patients usually present with chronic pelvic pain, dysmenorrhea, dyspareunia, and infertility. Endometriosis-related pain has an adverse effect on the quality of life of these patients [3,4]. Endometriomas are diagnosed through the use of abdominal and/or transvaginal ultrasound (TVUS) by their smooth ground glass echogenicity [5]. Magnetic resonance imaging (MRI) contains homogeneous, hyperintense areas on T1-weighted images and low- and high-signal areas on T2-weighted images. This situation, which is defined as shadowing because of low signal areas, is because of the high viscosity of the cyst content, protein concentration, and iron accumulation because of recurrent hemorrhages, which cause a shortening in T2 [6].

According to the European Society of Human Reproduction and Embryology (ESHRE) (2022) guidelines, the most commonly prescribed treatments for endometriosis include drugs that alter the hormonal milieu, either by suppressing ovarian activity or by acting directly on steroid receptors and enzymes present in lesions. These drugs include progestogens, antiprogestogens, combined oral contraceptives (OCPs), gonadotropin-releasing hormone (GnRH) agonists, GnRH antagonists, levonorgestrel intrauterine devices (LNG-IUD), danazol, and aromatase inhibitors. All these hormone treatments lead to a clinically significant reduction in pain compared with a placebo [1]. Dienogest is a new generation of progestin carrying the pharmacological specialties of 19-norprogestin and progesterone derivatives. It has been shown that dienogest has strong progestogen, androgenic, mineralocorticoid, and glucocorticoid effects [1,6].

In the present study, we aimed to assess the effect of dienogest treatment on endometrioma size and endometriosis-related pain symptoms by evaluating the change in endometrioma volume utilizing TVUS and evaluating the pain scores of the same patients using a visual analogue scale (VAS) over a six-month follow-up period.

## Materials and methods

This prospective observational study was conducted at the Department of Obstetrics and Gynecology, University of Health Sciences Turkey, Istanbul Kanuni Sultan Suleyman Training and Research Hospital, between March 2020 and March 2021. The study protocol was approved by the institution’s ethics committee (19/02/2020_42). All patients gave their written informed consent before enrollment.

In total, 81 patients, aged 17-49, who were referred to our tertiary centre during the above-mentioned study period because of the presence of unilateral or bilateral endometriomas were included. Further inclusion criteria were the absence of hormone-dependent cancers such as breast cancer and the absence of any medical conditions contraindicating the onset of hormonal treatment such as active venous thromboembolism, previous or current cardiovascular disease, diabetes with cardiovascular complications, current severe liver disease, and pregnancy. Out of these 81 patients, two were treated with laparoscopic removal of their endometriomas without receiving any medical treatment; thus, they were excluded. An additional three were excluded because of their fertility wishes and were referred for fertility treatment. A total of 76 patients received dienogest treatment. However, eight patients discontinued their treatment because of drug-related metrorrhagia, three because of secondary amenorrhea, and one because of mood changes. In total, 12 patients discontinued their treatment, for a discontinuation rate of 15.79%, and 64 patients were included in the final statistical calculations.

Patients’ demographic characteristics, including age, body mass index (BMI), gravidity, parity, fertility status, and fertility desire, were recorded before treatment. During the initial visit, each patient underwent a thorough gynaecological examination with TVUS. Ovarian endometrioma was diagnosed using TVUS performed by experienced sonographers utilizing "pattern recognition" through subjective evaluation of grayscale and Doppler-ultrasound characteristics. The diagnosis of the endometrioma was made according to International Ovarian Tumour Analysis (IOTA) rules when "an adnexal mass with ground glass echogenicity of the cyst fluid, one to four locules, and no papillations with detectable blood flow" was observed [7]. Endometrioma diameters were measured by taking the mean of the largest horizontal and vertical components. The endometrioma size was recorded in millimeters. Bilateral endometriomas were measured separately, and their mean was recorded. Serum CA-125 levels were determined before the onset of treatment and recorded. In cases of uncertain cyst appearance on TVUS and/or high levels of CA-125, patients received MRI imaging, and their findings were evaluated by a gynaecological oncologist and radiologist to rule out malignancy. Pain symptoms were also evaluated at the initial consultation, where patients’ dysmenorrhea and dyspareunia were assessed using VAS scores from 0 to 10, where 0 implies no pain and 10 implies severe pain. Each patient received dienogest treatment 2 mg/daily for six months. Follow-up visits were evaluated after three and six months of treatment to assess modifications in the patients’ pain symptoms and size. Endometrioma sizes and pain scores were assessed at each visit.

Data analysis was performed using SPSS (version 20.0; SPSS Inc., Chicago, IL, USA). Continuous variables are presented as the mean ± standard deviation (SD), and categorical variables are presented as percentages (%). Endometrioma sizes and VAS scores were recorded before the onset of treatment and at three- and six-month follow-ups and were compared using a paired t-test or the Wilcoxon signed-rank test.

## Results

Out of the 76 patients who received treatment with dienogest, 64 were compliant with their therapy over the six-month follow-up period. Hence, they were included in the analyses. The mean age was reported to be 33.9 ± 8.7 and the mean BMI was measured at 23.3 ± 3.3 (Table 1). When the infertility status of the patients was questioned, 15.6% of the patients were infertile, 68.8% were fertile, and 15.6% did not yet have fertility claims. The demographic variables are shown in Table 1.

**Table 1:** Patients’ demographic characteristics BMI: Body mass index

Demographic characteristics		Patients (N = 64) (mean + SD)	Min-Max
Age		33.9 ± 8.7	17-49
Gravidity		1.4 ± 1.3	0-5
Parity		1.2 ± 1.1	0-5
BMI		23.3 ± 3.3	18.3-31.6
CA-125 (IU)		80.7 ± 15.6	9-126.7
Endometrioma localization n(%)	Right unilateral	25 (39.1%)	
	Left unilateral	26 (40.6%)	
	Bilateral	13 (20.3%)	
Infertility n(%)	Yes	10 (15.6%)	
	No	44 (68.8%)	
Fertility wish (%)		10 (15.6%)	

**Figure 1:**
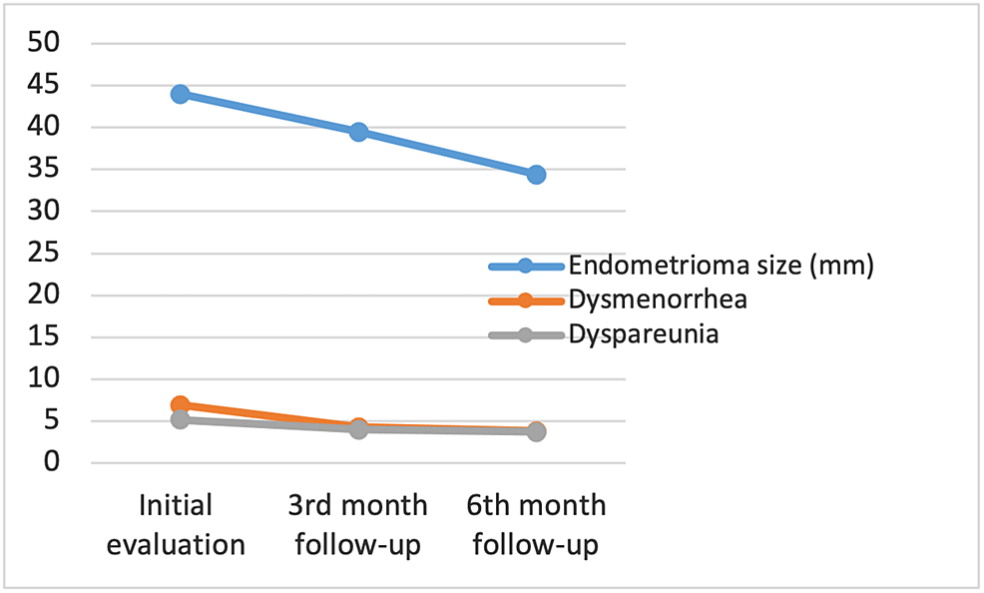
Graphical presentation of change in endometrioma size, dysmenorrhea and dyspareunia under the treatment of dienogest over six-month follow-up period

The mean endometrioma size before the onset of dienogest treatment was found to be 44.0 ± 13 mm (Table 2). The mean sizes were evaluated at the three- and six-month follow-ups and found to be 39.5 ± 15 mm and 34.4 ± 18 mm, respectively. A decrease in the mean size of 4.3 mm (10%) was found over the first three months of treatment with dienogest (Table 3). This decrease was observed to be 9.4 mm (21.4%) over the six months of the treatment period. When compared with the initial mean endometrioma size, the decrease in size was significant at both follow-ups (p<0.01). Figure 1 depicts the change in endometrioma size over the six-month follow-up period. Endometriomas were not detected with TVUS in 10 patients at the six-month follow-up.

**Table 2:** Comparison of mean endometrioma size, dysmenorrhea and dyspareunia VAS scores between initial assessment and 3rd and 6th month follow-up consultations p-value: p value of comparison between initial assessment and 3^rd^ month follow-up; p’-value: p value of comparison between initial assessment and 6^th^ month follow-up; p*-value: p value of comparison between 3^rd^ and 6^th^ month follow-ups. VAS: visual analogue scale.

Assessed variables	Initial evaluation (mean +SD)	3^rd^ month follow-up (mean +SD)	6^th^ month follow-up (mean +SD)	p-value	p’-value	p*-value
Endometrioma size (mm)	44.0 ± 13	39.5 ± 15	34.4 ± 18	<0.01	<0.01	<0.01
Dysmenorrhea	6.9 ± 2.6	4.3 ± 2.8	3.8 ± 2.7	<0.01	<0.01	0.12
Dyspareunia	5.2 ± 3.8	4.0 ± 3.2	3.7 ± 3.1	<0.01	<0.01	0.38

**Table 3:** Treatment dependent change in endometrioma size in mm and %

Endometrioma size assessment	Mean	p-value
3-month change in endometrioma size (mm)	−4.3	<0.01
3-month change in endometrioma size (%)	−10	<0.01
6-month change in endometrioma size (mm)	−9.4	<0.01
6-month change in endometrioma size (%)	−21.4	<0.01

Dysmenorrhea was evaluated using VAS scores at the initial consultation, the three-month follow-up, and the six-month follow-up (Table 2). The mean VAS score at the initial assessment was calculated as 6.9 ± 2.6. This value decreased to 4.3 ± 2.8 at the three-month follow-up, and a further decrease was calculated at the six-month follow-up (3.8 ± 2.7). When the VAS scores for dysmenorrhea were compared between the initial visit and the three- and six-month follow-ups, the decrease in mean VAS scores was significant (p<0.01). A 37.6% decrease in the mean VAS score was observed in the third month of treatment and a 44.9% decrease over the six months. However, the decrease between the third and sixth months was not statistically significant (p* = 0.38).

Dyspareunia was also evaluated as an important endometriosis-related pain symptom. At the initial assessment, the mean VAS score for dyspareunia was calculated as 5.2 ± 3.8. At the three-month evaluation, the mean score was 4.0 ± 3.2, and at the six-month assessment, a mean score of 3.7 ± 3.1 was reported. The decrease over the first three months was calculated at 23.07%, and over the six months of treatment, it was calculated at 28.8%. Similar to dysmenorrhea, a significant difference was observed between the initial pain scores and the three- and six-month follow-up scores, with p-values of <0.01 and <0.01, respectively. However, the difference between the three- and six-month follow-ups was not significant (7.5%, p-value of 0.12) (Figure 1).

## Discussion

Among progestogens, dienogest is a fourth-generation selective progestin that provides a significant local effect on endometriotic lesions. Because of their better profile in terms of tolerability, safety, and efficacy, progestin-only pills, among them dienogest, are the most suitable choice for the medical treatment of symptomatic endometriosis and to reduce the size of endometriomas. In symptomatic patients, the symptoms usually guide the choice of treatment, and the management options are medical and/or surgical. The presence of endometriomas and surgical management of them have a damaging effect on ovarian function and their reserve, along with a high rate of endometrioma recurrence after their removal [1,5,8]. These facts create a debate on ideal management in favour of nonsurgical treatment models.

In the present study, our aim was to evaluate the short-term effect of 2 mg/day Dienogest treatment on patients with endometriomas by assessing endometriosis-related dysmenorrhea and dyspareunia using the VAS scoring system and by evaluating endometrioma sizes with the TVUS. We reported a gradual decrease in the mean endometrioma size at the three- and six-month follow-up periods compared with the initial/pretreatment assessment. A decrease of 4.3 mm (10%) in the mean size was observed over the first three months of treatment with dienogest. This decrease was measured at 9.4 mm (21.4%) over the six months of the treatment period. When compared with the initial mean endometrioma size, the decrease in size was significant in both groups. Additionally, there was a decrease in the mean VAS score of dysmenorrhea at the three- and six-month follow-ups compared with the pretreatment assessment; however, the decrease between three and six months was not significant. Similar results were obtained for the mean VAS score of dyspareunia. In light of these findings, we have demonstrated a prompt onset of the positive effects of dienogest treatment both on endometrioma size and pain symptoms, which can already be observed after a three-month treatment period, and the continuation of these effects over the first six months of treatment.

A progressive decrease in the size of the endometrioma in patients taking 2 mg dienogest daily for at least six months has been reported [9-14]. Del Forno et al. showed that the mean diameter of endometriomas decreased by 2.51 mm after six months of dienogest treatment (2 mg/day) in 69 patients [11]. In a study of 63 patients conducted by Vignali et al., a 66.71% decrease in the mean endometrioma volume was calculated. Furthermore, Adnan and Aizzi measured the longest endometrioma diameter as 5.4 cm at the beginning, while it was 3.1 cm at the end of three months of dienogest treatment in 20 patients [14]. In our study, while the mean endometrioma size was 4.4 cm at the beginning, it was measured as being 3.95 cm at the end of three months and 3.44 cm at the end of six months. Although all studies reported a decrease in endometrioma size with the dienogest treatment, the discrepancy in the reported reduction could be because of the different methodologies utilized in the measurements of endometriomas. For example, Vignali et al. utilized three-dimensional measurements, whereas we conducted a two-dimensional measurement. Therefore, in future studies, standardization is needed in the endometrioma measurement to determine the precise effect of dienogest on the size of the endometrioma. Additionally, Adnan et al. reported that after three months of treatment, endometriomas disappeared in 18 out of 20 patients [14]. In our study, in 10 out of 64 patients, endometriomas disappeared after six months of treatment. A high rate of disappearance by Adnan et al. was not detected in our study, which could be because of our larger patient cohort.

Lang et al. studied endometriosis-associated pelvic pain (EAPP) in 255 Chinese women aged 18-45 years with laparoscopically diagnosed endometriosis [15]. They showed that with 2 mg/day dienogest, the mean VAS score of EAPP decreased from 57.6 mm to 18.9 mm over six months, resulting in an absolute difference of −38.7 mm. The greatest decrease in VAS score occurred in the first three months, and pain relief continued in the second three-month period. After 24 weeks of treatment, the difference between treatment arms for the mean reduction in EAPP was statistically significant in favor of dienogest (−24.54 mm) [15]. In our study, an absolute decrease of −2.6 was calculated for the mean VAS score of dysmenorrhea in the first three months and −3.1 at the end of the six months. The mean dyspareunia VAS score had an absolute decrease of −1.2 during the first three months and −1.5 over the entire six months of the follow-up period. Similar to the results by Lang et al., the decrease in VAS scores occurred predominantly in the first three months of treatment.

In another study, the effect of dienogest on pelvic pain was evaluated by Strowitzki et al. [16]. A total of 252 women were randomized to treatment with dienogest (2 mg/day, orally) or leuprolide acetate (LA) (3.75 mg, depot i.m. injection, every four weeks), with a follow-up period of six months. Absolute reductions in VAS scores from baseline to week 24 were 47.5 mm with Dienogest and 46.0 mm with LA, demonstrating the equivalence of Dienogest treatment in relation to LA. Hypoestrogenic effects (e.g., hot flushes) were reported less frequently in the dienogest group. They concluded that dienogest 2 mg/day orally demonstrated equivalent efficacy to depot LA at a standard dose in relieving the pain associated with endometriosis while offering advantages in safety and tolerability [16,17].

In addition to both progestin-only pills containing dienogest 2 mg and cyclic OCP, GnRH analogs, and aromatase inhibitors with/without norethindrone acetate were studied to assess their effects on endometrioma-related symptoms. A reduction in endometrioma volume was reported with GnRH analogues (up to 51%) and aromatase inhibitors (up to 75%) [18,19]. However, the associated hypoestrogenism has been reported as a major limitation in the long-term use of these therapies.

Our study has a few limitations. The observational nature of its design does not allow for randomization, which is one of its major limitations. However, to minimize selection bias, all patients who visited the clinic during the study period and met the inclusion criteria were recruited consecutively. Another limitation was the cohort size. Although our cohort was not large, it was comparable in size to the available studies, allowing for the interpretation of results in a statistically significant manner. Additionally, as mentioned above, some studies used three-dimensional measurements to calculate endometrioma sizes; some studies used the maximum diameter; however, we utilized two-dimensional measurements to decrease the sonographer-dependent errors. Future randomized studies with larger cohorts and standardized measurement techniques could help in determining the side-effect profile of dienogest treatment and the reasons for treatment discontinuation, and longer follow-up periods could allow clinicians to draw conclusions on the long-term effects of the treatment and design treatment plans accordingly.

## Conclusions

Based on our results, we can conclude that dienogest, which is a new generation of progestin, is an effective agent in the treatment of endometriomas in its ability to alleviate endometriosis-related dysmenorrhea and dyspareunia and in reducing the size of the endometriomas. Dienogest can be one of the first choices in the treatment of endometriomas, especially in patients with a fertility desire where ovarian reserve and the protection of ovarian tissue are critical.

## References

[1] Becker CM, Bokor A, Heikinheimo O (2022). ESHRE guideline: endometriosis. Hum Reprod Open.

[2] Hediger ML, Hartnett HJ, Louis GM (2005). Association of endometriosis with body size and figure. Fertil Steril.

[3] Bhatt S, Kocakoc E, Dogra VS (2006). Endometriosis: sonographic spectrum. Ultrasound Q.

[4] De Graaff AA, D'Hooghe TM, Dunselman GA, Dirksen CD, Hummelshoj L, Simoens S (2013). The significant effect of endometriosis on physical, mental and social wellbeing: results from an international cross-sectional survey. Hum Reprod.

[5] Webb EM, Green GE, Scoutt LM (2004). Adnexal mass with pelvic pain. Radiol Clin North Am.

[6] Bis KG, Vrachliotis TG, Agrawal R, Shetty AN, Maximovich A, Hricak H (1997). Pelvic endometriosis: MR imaging spectrum with laparoscopic correlation and diagnostic pitfalls. Radiographics.

[7] Timmerman D, Ameye L, Fischerova D (2010). Simple ultrasound rules to distinguish between benign and malignant adnexal masses before surgery: prospective validation by IOTA group. BMJ.

[8] Dunselman GA, Vermeulen N, Becker C (2014). ESHRE guideline: management of women with endometriosis. Hum Reprod.

[9] Momoeda M, Harada T, Terakawa N, Aso T, Fukunaga M, Hagino H, Taketani Y (2009). Long-term use of dienogest for the treatment of endometriosis. J Obstet Gynaecol Res.

[10] Sugimoto K, Nagata C, Hayashi H, Yanagida S, Okamoto A (2015). Use of dienogest over 53 weeks for the treatment of endometriosis. J Obstet Gynaecol Res.

[11] Del Forno S, Mabrouk M, Arena A, Mattioli G, Giaquinto I, Paradisi R, Seracchioli R (2019). Dienogest or Norethindrone acetate for the treatment of ovarian endometriomas: Can we avoid surgery?. Eur J Obstet Gynecol Reprod Biol.

[12] Angioni S, Pontis A, Malune ME (2020). Is dienogest the best medical treatment for ovarian endometriomas? Results of a multicentric case control study. Gynecol Endocrinol.

[13] Vignali M, Belloni GM, Pietropaolo G, Barbasetti Di Prun A, Barbera V, Angioni S, Pino I (2020). Effect of Dienogest therapy on the size of the endometrioma. Gynecol Endocrinol.

[14] Adnan H, Aizzi FJ (2017). Recurrent endometrioma; outcome of medical management with dienogest. Eur Exp Biol.

[15] Lang J, Yu Q, Zhang S (2018). Dienogest for treatment of endometriosis in Chinese women: a placebo-controlled, randomized, double-blind Phase 3 study. J Womens Health (Larchmt).

[16] Strowitzki T, Marr J, Gerlinger C, Faustmann T, Seitz C (2010). Dienogest is as effective as leuprolide acetate in treating the painful symptoms of endometriosis: a 24-week, randomized, multicentre, open-label trial. Hum Reprod.

[17] Strowitzki T, Faustmann T, Gerlinger C, Schumacher U, Ahlers C, Seitz C (2015). Safety and tolerability of dienogest in endometriosis: pooled analysis from the European clinical study program. Int J Womens Health.

[18] Taniguchi F, Enatsu A, Ota I, Toda T, Arata K, Harada T (2015). Effects of low dose oral contraceptive pill containing drospirenone/ethinylestradiol in patients with endometrioma. Eur J Obstet Gynecol Reprod Biol.

[19] Ferrero S, Remorgida V, Venturini PL, Leone Roberti Maggiore U (2014). Norethisterone acetate versus norethisterone acetate combined with letrozole for the treatment of ovarian endometriotic cysts: a patient preference study. Eur J Obstet Gynecol Reprod Biol.

